# Use of Prenatal Telehealth in the First Year of the COVID-19 Pandemic

**DOI:** 10.1001/jamanetworkopen.2023.37978

**Published:** 2023-10-10

**Authors:** Rebecca A. Gourevitch, Amylee Anyoha, Mir M. Ali, Priscilla Novak

**Affiliations:** 1 Department of Health Policy and Management, School of Public Health, University of Maryland, College Park

## Abstract

This cross-sectional study analyzes factors associated with the use of telehealth for prenatal care during the first year of the COVID-19 pandemic.

## Introduction

The COVID-19 pandemic prompted many prenatal care clinicians to incorporate virtual care. Studies^1,2,3^ at single clinical sites have found mixed results on prenatal telehealth access and satisfaction. However, few studies used data across multiple clinical sites. We leveraged a multistate representative survey to describe variation in prenatal telehealth use and reasons for its nonuse at the height of the pandemic.

## Methods

This cross-sectional study followed the STROBE reporting guideline and was determined exempt by the University of Maryland, College Park institutional review board because the data were deidentified. We used data from the 2020 Pregnancy Risk Assessment Monitoring System (PRAMS) across 29 states or localities (sites). The sample included respondents who answered the COVID-19 experiences questionnaire during site-months that achieved a 50% response rate (eTable and eFigure in Supplement 1). Participants provided informed consent through the survey.

Our primary outcome was a binary indicator for having had any prenatal telehealth visits (eMethods in Supplement 1). Respondents who did not use any prenatal telehealth were asked for the reasons, which we grouped into 4 secondary outcomes: personal preference, appointment availability, technological barriers, or other reason (eMethods in Supplement 1). See eMethods in Supplement 1 for details on included respondent demographic and health characteristics.

We used fully adjusted linear probability models to describe the association of prenatal telehealth use with respondent characteristics. In the subgroup of respondents who had no prenatal telehealth visits, we calculated unadjusted rates of the reasons. All analyses used 2-sided tests of statistical significance (*P* *<* *.*05), applied PRAMS’ survey weights, and were conducted using Stata statistical software version 16.1 (StataCorp). Data analysis was conducted from January to June 2023.

## Results

Our sample included 12 073 respondents who gave birth between June and December 2020 (weighted number, 628 473). A total of 6274 respondents (53%) had private insurance, 4904 (54%) were non-Hispanic White, and 9733 (87%) lived in urban counties (Table 1). All reported percentages are weighted.

**Table 1.** Respondent Characteristics by Use of Any Prenatal Telehealth

Characteristic	Respondents, No. (weighted %)^a^		
	All (N = 12 073)	Used no prenatal telehealth (n = 7686)	Used any prenatal telehealth (n = 4387)
Insurance type			
Private	6274 (53)	3870 (51)	2404 (57)
Medicaid	5048 (40)	3327 (42)	1721 (37)
Uninsured	291 (3)	190 (3)	101 (2)
Missing	460 (4)	299 (4)	161 (4)
Race and ethnicity^b^			
Asian or Pacific Islander, non-Hispanic	1034 (5)	556 (5)	478 (7)
Black, non-Hispanic	2041 (14)	1295 (14)	746 (14)
Hispanic	2583 (21)	1663 (20)	920 (21)
Indigenous, non-Hispanic	374 (1)	256 (1)	118 (1)
Missing	564 (3)	269 (3)	295 (3)
Other or multiple, non-Hispanic	573 (3)	342 (3)	231 (2)
White, non-Hispanic	4904 (54)	3305 (55)	1599 (52)
Educational level			
Less than high school	1340 (11)	871 (11)	469 (10)
High school	2933 (25)	1955 (26)	978 (22)
More than high school	7703 (63)	4797 (62)	2906 (67)
Missing	97 (1)	63 (1)	34 (1)
Marital status			
Married	7290 (61)	4515 (60)	2775 (64)
Not married	4778 (39)	3168 (40)	1610 (36)
Missing	5 (<1)	3 (<1)	2 (<1)
Rurality			
Urban	9733 (87)	5930 (84)	3803 (92)
Rural	1887 (12)	1347 (14)	540 (8)
Missing	453 (2)	409 (2)	44 (<1)
Age, y			
<20	451 (4)	316 (4)	135 (3)
20-24	2020 (17)	1396 (19)	624 (15)
25-29	3281 (28)	2143 (28)	1138 (27)
30-34	3782 (31)	2294 (29)	1488 (33)
≥35	2539 (21)	1537 (20)	1002 (22)
Missing	0	0	0
Parity			
Primiparous	5011 (41)	3096 (39)	1915 (43)
Multiparous	7042 (59)	4574 (60)	2468 (57)
Missing	20 (<1)	16 (<1)	4 (<1)
Prepregnancy depression			
No depression	10 007 (84)	6323 (84)	3684 (85)
Depression	1944 (15)	1292 (15)	652 (14)
Missing	122 (1)	71 (1)	51 (1)
Prepregnancy diabetes			
No diabetes	11 562 (97)	7361 (97)	4201 (96)
Diabetes	358 (2)	231 (2)	127 (2)
Missing	153 (1)	94 (1)	59 (1)
Prepregnancy hypertension			
No hypertension	11 198 (94)	7108 (94)	4090 (94)
Hypertension	740 (5)	502 (5)	238 (4)
Missing	135 (1)	76 (1)	59 (1)

^a^ Data are unweighted sample sizes and weighted percentages using survey weights from the 2020 Pregnancy Risk Assessment Monitoring System.

^b^ As listed on the survey instrument, Asian or Pacific Islander included respondents who were Chinese, Japanese, Filipino, Hawaiian, or “other Asian”; Indigenous included those who were American Indian or Alaska Native; and other or multiple included “other race” or “mixed race.”

Approximately 1 in 3 respondents used prenatal telehealth. Compared with respondents with private insurance, those with Medicaid had no adjusted difference in prenatal telehealth use, but uninsured respondents were 14.6 percentage points less likely to use prenatal telehealth (Table 2). Hispanic, Asian or Pacific Islander, and Indigenous respondents were more likely to use prenatal telehealth than non-Hispanic White respondents. Those in rural areas were less likely to use prenatal telehealth than urban respondents. Among the 7686 respondents who did not use prenatal telehealth, the most reported reason was a personal preference for in-person care (5470 respondents [70%]), followed by no appointment availability (1873 respondents [26%]), other reasons (1160 respondents [14%]), or technological barriers (490 respondents [5%]).

**Table 2.** Adjusted Differences in Use of Prenatal Telehealth by Respondent Characteristics

Characteristic	Adjusted coefficient, percentage points (95% CI)^a^
Insurance type	
Private	0 [Reference]
Medicaid	−1.9 (−5.1 to 1.3)
Uninsured	−14.6 (−22.1 to −7.2)
Race and ethnicity^b^	
Asian or Pacific Islander, non-Hispanic	7.7 (2.4 to 13.0)
Black, non-Hispanic	1.7 (−2.2 to 5.6)
Hispanic	7.1 (3.2 to 11.1)
Indigenous, non-Hispanic	17.5 (2.9 to 32.0)
Other, or multiple, non-Hispanic	−2.6 (−9.6 to 4.5)
White, non-Hispanic	0 [Reference]
Educational level	
Less than high school	0 [Reference]
High school	−2.2 (−7.0 to 2.7)
More than high school	−0.4 (−5.2 to 4.4)
Marital status	
Married	0 [Reference]
Not married	−0.1 (−3.3 to 3.1)
Rurality	
Urban	0 [Reference]
Rural	−9.5 (−13.0 to −5.9)
Age, y	
<20	0 [Reference]
20-24	6.4 (−0.7 to 13.5)
25-29	11.3 (4.1 to 18.5)
30-34	14.0 (6.6 to 21.4)
≥35	14.6 (6.9 to 22.3)
Parity	
Primiparous	0 [Reference]
Multiparous	−5.5 (−8.3 to −2.7)
Prepregnancy depression	
No depression	0 [Reference]
Depression	0.7 (−3.1 to 4.5)
Prepregnancy diabetes	
No diabetes	0 [Reference]
Diabetes	0.1 (−8.0 to 8.1)
Prepregnancy hypertension	
No hypertension	0 [Reference]
Hypertension	−2.3 (−8.2 to 3.6)

^a^ Results are from linear probability models controlling for the characteristics listed in the table. Coefficients are interpreted as the adjusted percentage point difference in likelihood of using prenatal telehealth, compared with the reference category (constant = 26.3).

^b^ As listed on the survey instrument, Asian or Pacific Islander included respondents who were Chinese, Japanese, Filipino, Hawaiian, or “other Asian”; Indigenous included those who were American Indian or Alaska Native; and other or multiple included “other race” or “mixed race.”

## Discussion

This cross-sectional study found that most respondents who gave birth between June and December 2020 did not use prenatal telehealth, and a personal preference for in-person care was the most common reason. Patients’ preferences should influence how prenatal telehealth, which has both benefits and drawbacks, is incorporated into their care.^4^

The similar rate of telehealth use between privately insured and Medicaid beneficiaries may be due to Medicaid programs’ efforts to enable telehealth access. Higher use among Indigenous respondents may be associated with the Indian Health System’s prepandemic telehealth infrastructure.^5^

Limitations include that the telehealth survey questions were not fielded after 2020 and did not inquire about quality of care or remote monitoring. Strengths include the use of multisite data; our results by insurance type and race and ethnicity are more consistent with national surveys across conditions than with single-site studies of prenatal telehealth,^6^ highlighting the importance of multisite research to inform best practices.

## Notes

Supplement 1. **eTable.** Sites Included in the Analytic Sample, by First Month of Inclusion **eFigure.** Construction of the Analytic Sample **eMethods.** Collection of Demographic Data and Construction of Primary Outcomes

Supplement 2.
Data Sharing Statement
