# Supplementary material for: Use of Prenatal Telehealth in the First Year of the COVID-19 Pandemic
Source: JAMA Netw Open. 2023 Oct 10;6(10):e2337978. doi: 10.1001/jamanetworkopen.2023.37978 (PMC10565607; doi:10.1001/jamanetworkopen.2023.37978)
Supplement: Supplement 1. — eTable. Sites Included in the Analytic Sample, by First Month of Inclusion eFigure. Construction of the Analytic Sample eMethods. Collection of Demographic Data and Construction of Primary Outcomes [file jamanetwopen-e2337978-s001.pdf]

Supplemental Online Content

Gourevitch RA, Anyoha A, Ali MM, Novak P. Use of prenatal telehealth in the first year of the COVID-19 pandemic. *JAMA Netw Open*. 2023;6(10):e2337978. doi:10.1001/jamanetworkopen.2023.37978

eTable. Sites Included in the Analytic Sample, by First Month of Inclusion
eFigure. Construction of the Analytic Sample
eMethods. Collection of Demographic Data and Construction of Primary Outcomes

This supplemental material has been provided by the authors to give readers additional information about their work.

© 2023 Gourevitch RA et al. *JAMA Network Open.*

eTable. Sites Included in the Analytic Sample, by First Month of Inclusion

Site	First Month of Inclusion in Sample
Alaska	July
Arkansas	October
Arizona	July
Connecticut	July
Washington, D.C.	July
Delaware	August
Florida	October
Georgia	October
Iowa	July
Illinois	July
Louisiana	July
Massachusetts	June
Maryland	July
Michigan	October
Missouri	July
North Dakota	October
Nebraska	August
New Jersey	July
New York City	July
Oregon	July
Pennsylvania	July
Puerto Rico	July
South Dakota	July
Tennessee	August
Utah	July
Virginia	August
Vermont	July
West Virginia	July
Wyoming	July

© 2023 Gourevitch RA et al. *JAMA Network Open.*

eFigure. Construction of the Analytic Sample

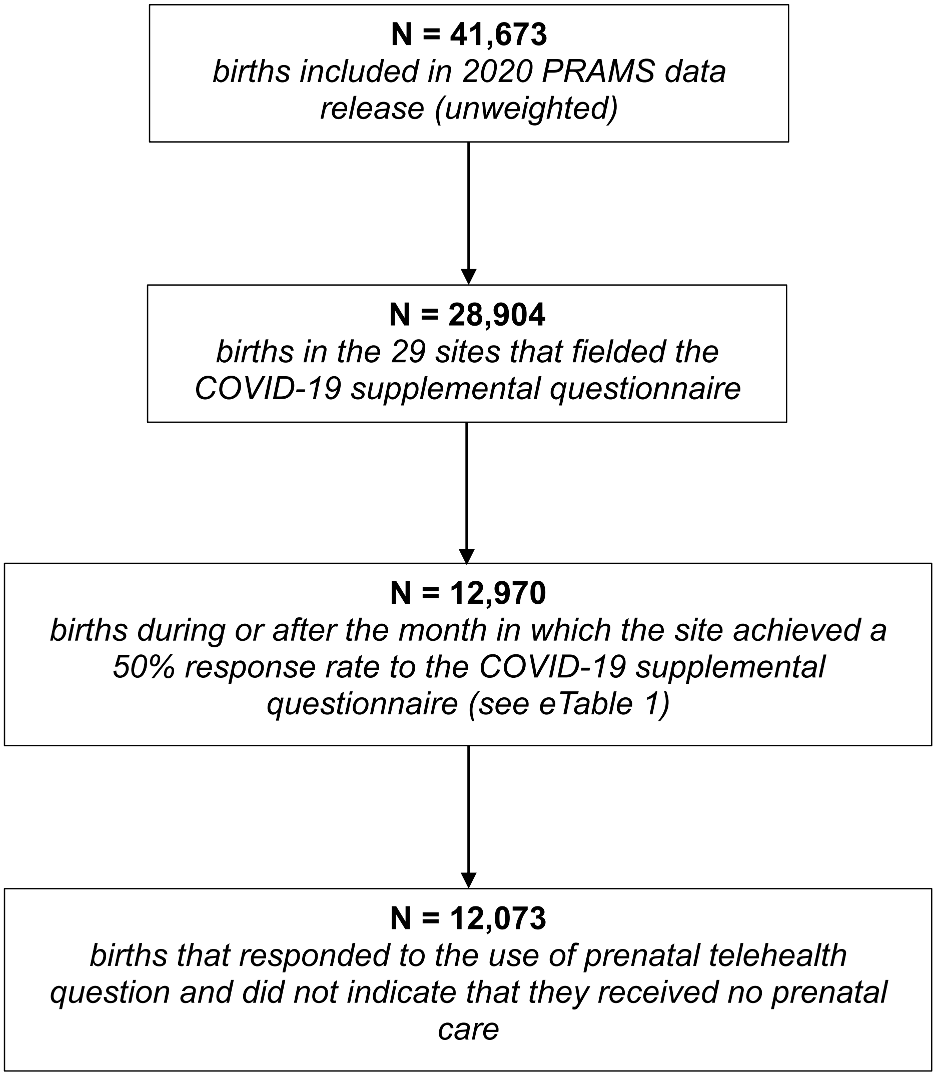

© 2023 Gourevitch RA et al. *JAMA Network Open.*

eMethods. Collection of Demographic Data and Construction of Primary Outcomes

We hierarchically classified respondents' self-reported prenatal insurance as Medicaid, private, or uninsured. We used the included birth certificate data to describe respondents' race and ethnicity, educational attainment, marital status, age, rural residence, and parity. We measured self-reported prepregnancy health (depression, diabetes, and high blood pressure or hypertension). Race and ethnicity, socially constructed categories that capture exposure to social determinants of health including structural and interpersonal racism, were included to be consistent with prior analyses of telehealth use.

CV1. During the COVID-19 pandemic, which types of *prenatal care* appointments did you attend?

**Response option**	**Primary outcome definition**
In-person appointments only	Did not use virtual care
Virtual appointments (video or telephone) only	Used virtual care
Both in-person and virtual appointments	Used virtual care
I did not have prenatal care	Not in analytic sample
Missing	Not in analytic sample

CV2. What are the reasons that you did not attend virtual appointments for *prenatal care*? For each one, check No if it was not a reason or Yes if it was.

	Personal preference	Appointment availability	Technologi-cal barriers	Other reason
Lack of availability of virtual appointments from my provider		X		
Lack of an available telephone to use for appointments			X	
Lack of enough cellular data or minutes			X	
Lack of a computer or device			X	
Lack of internet service or had unreliable internet			X	
Lack of a private or confidential space to use				X
I preferred seeing my health care provider in person	X			
Other reason (please tell us _____)				X

© 2023 Gourevitch RA et al. *JAMA Network Open.*
