# Supplementary material for: Use of Prenatal Telehealth in the First Year of the COVID-19 Pandemic
Source: JAMA Netw Open. 2023 Oct 10;6(10):e2337978. doi: 10.1001/jamanetworkopen.2023.37978 (PMC10565607; doi:10.1001/jamanetworkopen.2023.37978)
Supplement: Supplement 2. — Data Sharing Statement [file jamanetwopen-e2337978-s002.pdf]

Data Sharing Statement

Gourevitch. Use of Prenatal Telehealth in the First Year of the COVID-19 Pandemic. *JAMA Netw Open*. Published October 13, 2023. doi:10.1001/jamanetworkopen.2023.37978

Data
Data available: No

Additional Information
Explanation for why data not available: Our data use agreement does not permit the sharing of data. These data can be requested directly from PRAMS (at no cost).
